# Elevation of *Il6* is associated with disturbed let-7 biogenesis in a genetic model of depression

**DOI:** 10.1038/tp.2016.136

**Published:** 2016-08-16

**Authors:** Y B Wei, J J Liu, J C Villaescusa, E Åberg, S Brené, G Wegener, A A Mathé, C Lavebratt

**Affiliations:** 1 Neurogenetics Unit, Department of Molecular Medicine and Surgery, Karolinska Institutet, Stockholm, Sweden; 2 Center for Molecular Medicine, Karolinska Institutet, Stockholm, Sweden; 3 Neurogenetics Unit, Department of Molecular Biochemistry and Biophysics, Karolinska Institutet, Stockholm, Sweden; 4 Department of Neurobiology, Care Sciences and Society, Karolinska University Hospital Huddinge, Stockholm, Sweden; 5 Translational Neuropsychiatry Unit, Department of Clinical Medicine, Aarhus University, Aarhus, Denmark; 6 Centre of Excellence for Pharmaceutical Sciences, North-West University, Potchefstroom, South Africa; 7 Section for Psychiatry, Department of Clinical Neuroscience, Karolinska Institutet, Stockholm, Sweden

## Abstract

Elevation of the proinflammatory cytokine IL-6 has been implicated in depression; however, the mechanisms remain elusive. MicroRNAs (miRNAs) are small non-coding RNAs that inhibit gene expression post-transcriptionally. The lethal-7 (let-7) miRNA family was suggested to be involved in the inflammation process and IL-6 was shown to be one of its targets. In the present study, we report elevation of *Il6* in the prefrontal cortex (PFC) of a genetic rat model of depression, the Flinders Sensitive Line (FSL) compared to the control Flinders Resistant Line. This elevation was associated with an overexpression of LIN28B and downregulation of let-7 miRNAs, the former an RNA-binding protein that selectively represses let-7 synthesis. Also DROSHA, a key enzyme in miRNA biogenesis was downregulated in FSL. Running was previously shown to have an antidepressant-like effect in the FSL rat. We found that running reduced *Il6* levels and selectively increased let-7i and miR-98 expression in the PFC of FSL, although there were no differences in LIN28B and DROSHA expression. Pri-let-7i was upregulated in the running FSL group, which associated with increased histone H4 acetylation. In conclusion, the disturbance of let-7 family biogenesis may underlie increased proinflammatory markers in the depressed FSL rats while physical activity could reduce their expression, possibly through regulating primary miRNA expression via epigenetic mechanisms.

## Introduction

In the past two decades, clinical evidence has linked inflammatory responses with psychiatric disorders including major depressive disorder (MDD).^1, 2^ Cytokines, chemical messengers between immune cells, have been shown to have an important role in mediating behavioral, neuroendocrine and neurochemical features of MDD.^3^ Elevated levels of proinflammatory cytokines, such as interleukin-1β (IL-1β), tumor necrosis factor α (TNF-α) and IL-6 have been found in serum/plasma and cerebrospinal fluid of depressed patients, also in the absence of comorbid medical illness;^4, 5^ the most consistent result being an increase in IL-6.^5, 6, 7^ In addition, stimulation of the immune system with lipopolysaccharide can elicit symptoms of depression in humans with no previous episodes of depression.^8, 9^ Several findings also indicated that IL-6 has a pathophysiological role in depression, especially in patients who fail to respond to selective serotonin reuptake inhibitors.^6, 10, 11, 12, 13^ Physical exercise has been shown to have antidepressant effects and to reduce the risk for elevated levels of proinflammatory markers.^14, 15^ Thus, although accumulated evidence shows increased IL-6 in MDD, the mechanisms underlying these alterations have not been clarified.

MicroRNAs (miRNAs) are small non-coding RNAs that typically function as key post-transcriptional repressors of gene expression.^16^ MiRNAs control a variety of developmental and cellular processes and evidence has linked altered miRNA expression with psychiatric disorders, for example, MDD.^17, 18, 19^ The classic miRNA biogenesis begins with transcription of primary transcripts (pri-miRNAs) by RNA-PolII. In the cell nucleus, pri-miRNAs are processed by DROSHA and its cofactor DGCR8, releasing the 60–80 nucleotides (nt) precursors (pre-miRNAs). After transfer to the cytoplasm, pre-miRNAs are further cleaved by DICER to generate approximately 22 nt double-stranded mature miRNAs. One strand of the mature miRNA is incorporated into the RNA-induced silencing complex (RISC), whereas the other strand is degraded. The miRNA-RISC regulates target mRNA expression through mRNA degradation and/or translational repression.^16^

Lethal-7 (let-7) is one of the most studied miRNA families and is highly conserved between species.^20^ In human, the let-7 family consists of 12 genes encoding nine distinct miRNAs (let-7a to let-7i and miR-98). There is increasing evidence suggesting the involvement of the let-7 family in inflammation and immune response.^21, 22, 23^ A previous study showed that the let-7 family directly inhibited IL-6 expression in breast cancer cell lines, and thereby may act as an immunorepressor.^24^ Let-7 is abundant in adult brain and has been implicated in neuronal proliferation and differentiation and synaptic plasticity,^25, 26, 27, 28^ but it is not known whether it has a role in the pathophysiology of depression. In cancer research, coordinated downregulation of multiple let-7 family members was found in many tumor types.^23, 29, 30, 31^ This reduction was associated with an overexpression of LIN28 (including paralogous LIN28A and LIN28B in mammals), an RNA-binding protein that selectively represses let-7 maturation.^24, 32, 33^ Importantly, a recent study showed that LIN28B and LIN28A inhibited let-7 expression by different mechanisms, that is, LIN28B directly binds the primary let-7 (pri-let-7) transcripts and prevents DROSHA-mediated cleavage.^34^ Besides, heteronuclear ribonucleoprotein A1 (hnRNPA1) was shown to negatively regulate let-7 biogenesis in cells lacking LIN28 expression.^35^

The Flinders Sensitive Line (FSL) was selectively bred for increased sensitivity to the anticholinesterase agent diisopropyl fluorophosphate and exhibits behaviors that resemble a number of symptoms in human depression.^36, 37^ FSL and their controls, the Flinders Resistant Line (FRL) are widely used to explore putative pathophysiology of human depression and test antidepressant-like effects of both pharmacological and non-pharmacological interventions such as serotonin reuptake inhibitor,^38^ histone deacetylase inhibitor,^39^ deep brain stimulation,^40^ electroconvulsive stimuli^41^ and voluntary wheel running.^42^ Previously, we have shown altered expression of the inflammatory markers both centrally and in the periphery in the FSL depression model.^43, 44^ We hypothesized that *Il6* expression was elevated in the prefrontal cortex (PFC) of the FSL strain compared with FRL, and that this elevation would associate with a downregulation of the let-7 family, in turn influenced by alterations in miRNA biogenesis. Second, we hypothesized that physical exercise would lower the elevated *Il6* levels in the PFC of the FSL rats, by normalizing let-7 expression.

## Materials and methods

### Tissue samples and wheel running experiments

The PFC from 3-month-old male FRL/FSL rats (*n*=7 in each group) was dissected according to the method of Glowinski and Iversen^45^ and stored at −80 °C until subsequent analyses. Wheel running experiments were performed as previously described.^42^ In brief, 3-month-old male FSL rats were individually housed, randomly assigned with either free access (FSL-runner, *n*=7) or no access (FSL-control, *n*=7) to a running wheel during a period of 35 days. The sample sizes were chosen on the basis of previous publications and literatures in relevant field. Running data were sampled 48 times per day using a computer-based data system with customized software. The PFC region was dissected following wheel running experiments. The investigators were blinded to group allocation during all the procedures. All the animals had access to food and water *ad libitum*, and were subjected to a controlled 12-h light/dark cycle (lights on at 0700 h). All the experiments were approved by the Danish National Committee for Ethics in Animal Experimentation and the Ethical Committee for Protection of Animals at the Karolinska Institutet.

### RNA extraction and reverse transcription

Total RNA was extracted using miRNA Universal Kit (Qiagen, Hilden, Germany) followed by treatment with DNase I (Qiagen) to digest contaminating DNA. Concentrations were determined using the NanoDrop ND-1000 (NanoDrop Technologies, Waltham, MA, USA). Reverse transcription of miRNA was performed using Universal cDNA Synthesis Kit II for RT-PCR (Exiqon, Vedbaek, Denmark). UniSp6 RNA was spiked in for monitoring conversion efficiency. The reaction were incubated with the enzyme mix at 42 °C for 60 min followed by termination at 95 °C for 5 min. Complementary DNA (cDNA) to pri-miRNA and mRNA was synthesized using the SuperScript III First-Strand Synthesis System for real-time polymerase chain reaction (RT-PCR) (Invitrogen; Thermo Fisher Scientific, Waltham, MA, USA). In brief, equal amounts of RNA were random-hexamer primed at 25 °C for 10 min, followed by an incubation with SuperScript III RT at 50 °C for 50 min, and termination of the reaction at 85 °C for 5 min. cDNA of the miRNA and the mRNA was stored at −20 °C and RNA at −80 °C until further processing. All the experimental procedures were according to the manufacturer's protocols.

### Gene expression analyses

Amplification of cDNA corresponding to miRNAs, pri-miRNAs and mRNAs was assessed using RT-PCR. All RT-PCR amplifications were performed in triplicates using Power SYBR Green (Applied Biosystems; Thermo Fisher Scientific, Waltham, MA, USA) on an ABI PRISM 7900 HT Sequence Detection System (Applied Biosystems), with the following conditions: 95 °C for 10 min, followed by 40 repeats of 95 °C for 15 s (10 s for miRNA amplification), 60 °C for 1 min and a final dissociation stage to monitor amplification specificity. The microRNA LNA PCR primers (Exiqon) were used to amplify let-7 miRNA and the data were normalized to *Rnu5g*. Pri-let-7 and mRNA expression data were normalized to two reference genes (*Gapdh,* glyceraldehyde-3-phosphate dehydrogenase; and *Ppia,* cyclophilin A). Relative quantification was calculated using the qBase software (version 1.3.4).^46^ The tested genes and corresponding primer sequences are listed in Supplementary Table S1.

### Protein expression

Protein levels for LIN28B and DROSHA were quantified in naive FRL/FSL PFC using a modified western blot protocol, based on Lindfors *et al.*^47^ Briefly, following sample homogenization and centrifugation, the protein concentration of the lysates was measured using the Pierce BCA Protein Assay Kit (Thermo Fisher Scientific, Waltham, MA, USA). After denaturing at 95 °C for 5 min, equal amounts of protein (60 μg) were loaded on a NuPAGE Novex 4–12% Bis-Tris Gel (Invitrogen). The separated protein was transferred to Amersham Hybond ECL Nitrocellulose Membrane (GE Healthcare UK, Buckinghamshire, UK) for 1.5 h at room temperature, and then blocked with 5% nonfat milk for 1 h at room temperature. Immunoblotting was performed overnight at 4 °C with a polyclonal rabbit anti-LIN28B antibody (1:500 dilution; #5422, Cell Signaling Technology, Danvers, MA, USA) or with a polyclonal goat anti-DROSHA antibody (1:1000 dilution; ab58589; Abcam plc, Cambridge, UK) separately, and with a mouse monoclonal anti-β-actin antibody (1:10 000; A5316; Sigma-Aldrich, St Louis, MO, USA). After washing, the membrane for detecting LIN28B or DROSHA was incubated with HRP-linked goat anti-rabbit and sheep anti-goat secondary antibody, respectively (1:20 000 for LIN28B and 1:60 000 for DROSHA; Santa Cruz Biotechnology, Santa Cruz, CA, USA) and the membrane for detecting β-actin was incubated with HRP-linked goat anti-mouse secondary antibody (1:50 000; Santa Cruz Biotechnology) for 60 min at room temperature. Finally, immunoreactive bands were visualized using the Amersham ECL Plus Western Blotting Detection System (GE Healthcare), exposed to Amersham Hyperfilm ECL (GE Healthcare) and optical densities were quantified using the NIH ImageJ software (1.47 version). LIN28B and DROSHA expression levels were normalized to the expression levels of β-actin and the data were presented as relative quantifications.

### Chromatin immunoprecipitation

*In vivo* chromatin immunoprecipitation (ChIP) was performed according to Melas *et al.*^48^ Rat prefrontal cortex was homogenized and cross-linked with 1% formaldehyde for 10 min at room temperature followed by addition of glycine to stop the reaction. The samples were lysed and sonicated to shear the DNA into fragment sizes of 200–500 bp. Sonicated DNA was either aliquoted as genomic input DNA or immunoprecipitated using an anti-acetyl-histone H3 antibody (#06-599; EMD Millipore, Billerica, MA, USA), anti-acetyl-histone H4 antibody (#06-598; Millipore) or normal rabbit IgG (negative control; Millipore) at 4 °C overnight with rotation. Protein A Sepharose beads (Sigma-Aldrich) were used to catch the chromatin–antibody complexes. Immunoprecipitates containing protein–DNA complexes were washed and eluted followed by reverse crosslinking. DNA was purified using QIAquick PCR Purification Kit (Qiagen) and quantified by RT-PCR using Power SYBR Green (Applied Biosystems). PCR primers were designed to target within the pri-let-7i promoter (approximately 510–681 bp upstream of the putative transcription start site).^49^ The primer sequences are listed in Supplementary Table S1. The amount of immunoprecipitated pri-let-7i promoter DNA in each sample was calculated as percentage of genomic input DNA (% Input) as follows: % Input=2^−ΔCt sample^, where ΔCt sample=Ct (sample)−Ct (input).

### RNA immunoprecipitation

*In vivo* RNA immunoprecipitation (RIP) was performed using the Magna RIP RNA-Binding Protein Immunoprecipitation Kit according to the manufacturer's protocol (Millipore). In brief, rat PFC was lysed and immunoprecipitated overnight at 4 °C with a LIN28B antibody (3 μg per immunoprecipitation; #5422, Cell Signaling Technology) or normal rabbit IgG (negative control, 3 μg per immunoprecipitation; Millipore) bound to protein A/G magnetic beads. Ten percent of the lysate was saved as RIP input and stored at −80 °C until starting RNA purification. After washing, the immunoprecipitate was digested by proteinase K followed by RNA purification. cDNA was synthesized using the SuperScript III First-Strand Synthesis System for RT-PCR (Invitrogen) followed by RT-PCR for pri-let-7 expression. The primer sequences are listed in Supplementary Table S1.

### Statistical analyses

Data in the bar graphs are presented as mean values±s.e.m. Normality of the data and the homogeneity of the variance were tested using Shapiro–Wilk and Levene's tests, respectively. The difference in mean between two groups was assessed using two-tailed Student's *t* test. The threshold for statistical significance was set at *P*<0.05. All analyses were performed using IBM SPSS Statistics version 22 (IBM, Armonk, NY, USA).

## Results

### Increased *Il6* levels are associated with downregulation of let-7 family expression in the prefrontal cortex of the FSL rats

**Figure 1:**
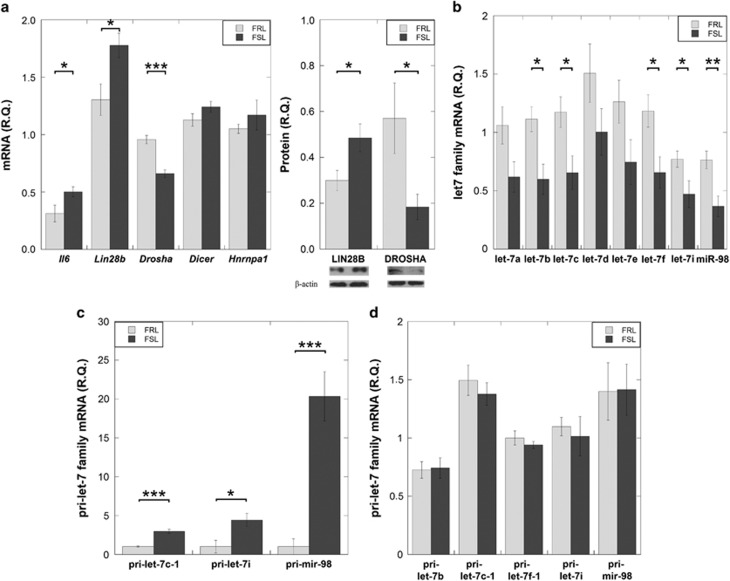
Gene and miRNA expression levels were measured in the prefrontal cortex of naïve FSL and FRL rats using RT-PCR. (**a**) Increased *Il6* and LIN28B, and decreased DROSHA levels in the FSL rats compared to FRL. (**b**) FSL rats exhibited decreased expression of several let-7 family members; Levels of let-7b, let-7c, let-7f, let-7i and miR-98 were significantly reduced. (**c**) RNA immunoprecipitation showed an enrichment of primary let-7 transcripts pri-let-7c-1, pri-let-7i and pri-mir-98 bound to LIN28B in FSL. (**d**) The reduction of mature let-7 was not associated with primary let-7 transcripts changes. Gene expression data are presented as relative quantifications (R.Q.), two reference genes (*Gapdh* and *Ppia*) were used for normalization of *Il6*, *Lin28b*, *Drosha* and the primary miRNA genes. *Rnu5g* was used for normalization of mature miRNAs. Lower panels in **a** (right) show representative western blot images of LIN28B and DROSHA from the same gel, respectively with β-actin as loading control. RIP data are presented as relative value to FRL. Data are presented as group means±s.e.m. For all figures: *n*=5–7 animals per group, *P<0.05, **P<0.01, ***P<0.001. FRL, Flinders Resistant Line; FSL, Flinders Sensitive Line; miRNA, microRNA; RIP, RNA immunoprecipitation; RT-PCR, real-time polymerase chain reaction.

First, we measured *Il6* mRNA levels in the PFC from FSL and FRL rats. The FSL had higher *Il6* mRNA levels compared to the FRL (*P*=0.045, Figure 1a). Second, we examined whether the increased *Il6* levels in the FSL associated with downregulation of let-7 expression. Several of the let-7 family members displayed reduced levels in the FSL compared to FRL. Specifically, let-7b, let-7c, let-7f, let-7i and miR-98 were significantly reduced in FSL (*P*=0.011, *P*=0.024, *P*=0.021, *P*=0.004 and *P*=0.006, respectively, Figure 1b). These results suggested that elevation of *Il6* in the FSL PFC is associated with a deficiency of let-7 family expression, possibly linked with a disturbed let-7 biogenesis.

### LIN28B elevation co-occurred with downregulation of the let-7 family in the FSL PFC

First, we tested the hypothesis that overexpression of LIN28A and LIN28B, acting as let-7 repressors, was associated with the let-7 family deficiency in the FSL. We found detectable levels of *Lin28b* mRNA but not of *Lin28a* (Ct>35) expressed in the PFC of adult FSL and FRL. Specifically, FSL rats exhibited increased LIN28B both at the mRNA and protein levels compared to the FRL (mRNA: *P*=0.021; protein: *P*=0.042, Figure 1a). We also tested whether *Hnrnpa1*, a negative regulator of let-7 that is independent of LIN28 regulation, was associated with the decreased let-7 expression in the FSL. *Hnrnpa1* expression levels in PFC were not different between the FSL and the FRL (*P*=0.38, Figure 1a). Second, to gain further support that LIN28B directly associated with pri-let-7 to block mature let-7 synthesis *in vivo*, we performed RIP analysis. The RIP results revealed that in the PFC of FSL there was an ~3 to 20-fold enrichment of the pri-let-7 transcripts (pri-let-7c-1, pri-let-7i, pri-mir-98) associated with LIN28B compared with the FRL (pri-let-7c-1: fold change 2.96, *P*<0.001; pri-let-7i: fold change: 4.43, *P*=0.019; pri-mir-98: fold change 20.34, *P*<0.001, respectively, Figure 1c). Third, we compared the total expression levels of pri-let-7b, pri-let-7c-1, pri-let-7f-1, pri-let-7i and pri-mir-98 between the FSL and FRL PFC; none of those pri-let-7 transcripts showed different expression between the rat strains (*P*>0.5, Figure 1d), indicating that reduction in mature let-7 expression in the FSL did not originate from decreased levels of pri-let-7 transcripts. Fourth, we assessed whether the expression levels of key enzymes (*Drosha* and *Dicer*) involved in miRNA biogenesis could potentially influence let-7 expression. The DROSHA levels were significantly reduced in the FSL compared with FRL (mRNA: *P*<0.001 and protein: *P*=0.032; Figure 1a). Thus, the downregulation of let-7 family members in FSL PFC region was associated with increased LIN28B levels, increased LIN28B binding to pri-let-7 transcripts and reduced DROSHA levels.

### Physical activity reduced *Il6* levels and rescued let-7 expression in the FSL PFC

**Figure 2:**
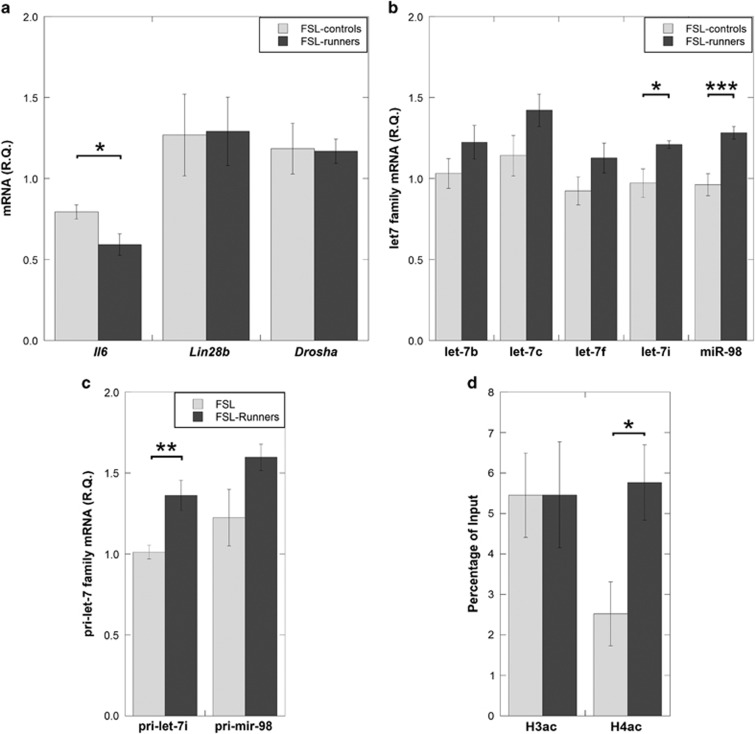
Gene and miRNA expression levels were measured in the prefrontal cortex of FSL with/without access to running wheel (FSL-runners versus FSL-controls) using RT-PCR. (**a**) Physical activity normalized *Il6* levels in the FSL-runner group but had no effect on *Lin28b* and *Drosha* mRNA levels. (**b**) In line with an *Il6* reduction in the FSL rats that were running, let-7i and miR-98 showed significantly increased expression. Specifically, upregulation of let-7i was associated with primary let-7i overexpression (**c**). (**d**) Chromatin immunoprecipitation (ChIP) showed an increased histone H4 acetylation (H4ac) but not histone H3 acetylation (H3ac) within the pri-let-7i promoter of the FSL-runners. Gene expression data were presented as relative quantifications (R.Q.), two reference genes (*Gapdh* and *Ppia*) were used for normalization of *Il6*, *Lin28b*, *Drosha* and the primary miRNA genes. *Rnu5g* was used for normalization of mature miRNAs. ChIP data are presented as percentage of genomic input DNA. Data are presented as group means±s.e.m. For all figures: *n*=5–7 animals per group, *P<0.05, **P<0.01, ***P<0.001. FSL, Flinders Sensitive Line; mRNA, messenger RNA; miRNA, microRNA; RT-PCR, real-time polymerase chain reaction.

First, we investigated where physical activity (voluntary wheel running) could normalize *Il6* expression in the FSL PFC. FSL rats in the running group (FSL-runners) had significantly lower *Il6* levels compared with the FSL rats without access to the running wheel (FSL-controls; *P*=0.025, Figure 2a). Second, we examined whether the *Il6* reduction in the FSL runners associated with upregulation of let-7 miRNAs that showed a difference between naïve FSL and FRL (let-7b, let-7c, let-7f, let-7i and miR-98). Let-7i and miR-98 levels were significantly increased in the FSL-runners compared with FSL-controls (*P*=0.026 and *P*=0.002, Figure 2b). No differences were found for the mRNA levels of *Lin28b* and *Drosha* (*P*>0.8), suggesting that the physical activity increased expression of certain let-7 miRNAs expression independently of LIN28B and DROSHA. Third, we examined whether increased let-7i and miR-98 in FSL-runners associated with an upregulation of pri-let-7i and pri-mir-98 transcripts. Pri-let-7i levels were significantly increased in the FSL-running group, and pri-mir-98 levels displayed a tendency for increased levels in the FSL-runners (*P*=0.004 and *P*=0.093 respectively, Figure 2c). In accord with the pri-let-7i upregulation, FSL-runners showed an increased histone H4 acetylation (*P*=0.030) but not histone H3 acetylation (*P*=0.99) within the pri-let-7i promoter (Figure 2d). Thus, physical activity reduced the elevated *Il6* levels in PFC region of the FSL rats and increased expression of certain let-7 family members present already at primary transcript stage, possibly through epigenetic mechanisms.

## Discussion

In the present study, we demonstrate increased *Il6* levels and in parallel decreased levels of let-7 miRNA family in the PFC of a well-established model of depression, the FSL rat.^36, 37^ Since the let-7 family is known to target *Il6*, the results suggest that the let-7 family dysregulation contributes to the overexpression of *Il6* in the PFC of FSL. The results also suggest that this decrease of let-7 miRNA levels is in part due to a disturbed LIN28B-mediated miRNA biogenesis, and possibly in part due to a dysregulated DROSHA. In addition, we show that physical activity normalizes *Il6* levels and could rescue let-7 expression. The let-7 upregulation by physical activity appeared not to be associated with miRNA biogenesis processes but rather with epigenetic changes upstream pri-let-7 mRNA.

### Elevation of *Il6* is associated with downregulation of let-7 miRNAs in the PFC of FSL, a rat model of depression

Elevation of peripheral proinflammatory cytokine IL-6 has been reported in clinical depression by a number of studies.^5, 6, 7^ Also, a recent report demonstrated increased IL-6 levels in the brain from rats that exhibited a depression-like phenotype after chronic stress paradigms.^10^ In line with these results, we found upregulation of *Il6* in the PFC of the FSL. However, according to our previous study, serum IL-6 levels were not different between the FSL and FRL.^44^ The let-7 family was previously shown to directly inhibit IL-6 expression in breast cancer cell lines, to be abundant in the adult brain and to be implicated in the regulation of neural stem cell proliferation, differentiation and synaptic plasticity.^22, 23, 24, 25, 26^ We showed that the *Il6* elevation in PFC of FSL was associated with a reduced let-7 miRNAs expression. Thus, five of the eight studied mature let-7 family members were significantly downregulated in the PFC, and the two of the remaining three showed a tendency to downregulation (let-7a: *P*=0.058; let-7e: *P*=0.080). The individual let-7 family members may compete with each other when exerting their repressive function since each member uses the same seed (5′-GAGGUA-3′ sequence of the let-7) as a template for recognizing complementary sites in the 3′-untranslated region of *Il6* (5′-UACCUCA-3′). Thus we observed that *Il6* correlated negatively with each of the let-7 family miRNAs, however, none with statistical significance. The let-7 family has an important role in early neurodevelopment. For example, let-7b and let-7d inhibit neural stem cell proliferation and promote differentiation in embryonic brains and adult neural stem cells.^28, 50^ However, other roles of let-7 family in the adult brain have been less investigated although let-7 is upregulated in later developmental stages and is one of the most abundant miRNA families in the adult brain.^51, 52^ We found that let-7 expression in the PFC of depressed FSL rats associated with elevated *Il6*. It is possible that let-7 dysregulation can lead to disturbances also in other pathophysiological processes because miRNAs often have multiple target genes. Let-7 was suggested to have a role in stress response and was shown to be decreased in distinct brain regions in two rat models of stress.^53^ Notably, let-7 expression appeared to be more decreased by chronic than acute stress paradigm,^53^ which may be in line with our data, as FSL is a genetic model that exhibits a persistent depression-like behavior.

### Let-7 deficiency is associated with LIN28B overexpression in the PFC of FSL rats

LIN28 is an RNA-binding protein that selectively represses let-7 maturation.^24, 32, 33^ Hypothalamic LIN28A and LIN28B levels are high in neonates but LIN28A declines sharply after birth, whereas LIN28B is maintained at a moderate level. Let-7 expression showed the opposite expression changes over time, possibly dependent on the LIN28 dynamics.^54^ In agreement, we found that *Lin28b* mRNA, but not *Lin28a,* was expressed at detectable levels in adult rat PFC, suggesting that LIN28B is the major paralog in regulating let-7 synthesis in the PFC. Specifically, the FSL displayed significant overexpression of LIN28B both at mRNA and protein levels compared with the FRL rats. Noteworthy, the let-7 deficiency in the FSL was not associated with changes in *Hnrnpa1*, which was a negative regulator of let-7 biogenesis when LIN28 regulation was absent.^35^ Next, we showed that LIN28B overexpression was associated with enrichment of LIN28B-pri-let-7 binding in FSL *in vivo*, which most likely led to excessive repression of mature let-7 synthesis, explaining the reduced mature let-7 levels. Let-7 can also inhibit LIN28B translation by binding to the 3′-untranslated region target sites, creating a double-negative feedback loop.^27^ Noteworthy, let-7 could also be regulated in an LIN28B-independent fashion, for example, through epigenetic mechanisms such as DNA methylation and histone modifications,^49, 55^ which is supported by our data from FSL rats under physical exercise (discussed in the next section). In addition to LIN28B changes, we observed that FSL PFC had a decreased DROSHA expression, suggesting a disturbed miRNA biogenesis probably not only in let-7 but also in a variety of other miRNAs. In line with this hypothesis, a recent study has demonstrated a general reduction of miRNA expression in the PFC from depressed suicidal subjects.^56^

### Physical activity rescued let-7 expression independent of LIN28B regulation

Previous studies demonstrated that physical activity in the form of wheel running exerts antidepressant-like effect and affects histone acetylation levels in the FSL rat.^42, 48^ In the present study, we found that FSL runners had reduced *Il6* levels in the PFC compared with FSL rats without access to a running wheel. This *Il6* decrease associated with an increased let-7 expression. Consistently, a recent study reported that physical exercise was able to induce let-7 in the mouse hippocampus.^57^ These results suggest that the antidepressant-like effect of physical activity may, in part, be due to brain let-7 expression. In agreement, let-7 miRNAs were also found to respond actively to antidepressant drug treatment. For instance, in a rat model of learned helplessness, enoxacin exerted an antidepressant-like effect coupled with a let-7a elevation in the frontal cortex.^58^ Further, in blood samples from depressed patients, a number of let-7 family members were upregulated after a 3-month treatment with escitalopram.^59^ Collectively, these results may suggest a role for let-7 as a therapeutic target in depression. We showed that the exercise-induced upregulation of let-7 miRNAs in FSL was independent of *Lin28b* and *Drosha* changes, implying that other mechanisms are involved in regulating let-7 expression in response to physical activity. In agreement with an effect upstream the primary miRNA transcripts, we found pri-let-7i transcript to be upregulated in the FSL runners, which was associated with an increase in histone H4 acetylation. Supported by ours and others' findings, physical activity increased histone acetylation in *Npy* and *Bdnf* genes and decreased histone deacetylase expression in the brain,^48, 60^ suggesting that pri-let-7i may share an upstream epigenetic regulation with stress resilience genes that is independent of LIN28B processing.

### Cholinergic dysregulation in the FSL rats

The FSL rats have increased sensitivity to cholinergic agonists compared with the control line FRL, which is associated with the increased density of muscarinic acetylcholine receptors and α_4_β_2_ nicotinic acetylcholine receptors, but not of α_7_ nicotinic acetylcholine receptors in the FSL brain,^61^ the α_7_ nicotinic receptor is implicated in the production of cytokines including IL-6.^62^ Consistent with the previous report,^61^ we found no statistically significant difference in expression level of *Chrna7* encoding α_7_ nicotinic receptor in the PFC between FSL and FRL (FSL: 0.89±0.13, FRL: 0.94±0.13, P=0.79; Relative quantification using RT-PCR, data are presented as group means±s.e.m.). This suggests that the elevated *Il6* levels in the FSL PFC was not due to an α_7_ nicotinic receptor disturbance. Moreover, previous studies demonstrated that the acetylcholinesterase activity was similar in FSL and FRL suggesting that the increased cholinergic sensitivity in the FSL was not due to the changes in the acetylcholinesterase activity.^63, 64^ However, one should keep in mind that alternative splicing and expression of *AChE,* encoding acetylcholinesterase, is regulated also epigenetically induced by external exposures. For example, the histone deacetylase inhibitor sodium butyrate, known to have an antidepressant effect,^39^ affected histone modifications that regulated *AChE*.^65^

### Limitations

There are limitations of this study: (1) Only one reference gene was used for the miRNA expression analysis: *Rnu5g*. The results from the FRL/FSL rats were consistent when adding also reference gene *U6*. However, as *U6* was affected by physical activity, it was not used as reference in this report. (2) IL-6 protein levels were not studied due to the lack of material. (3) The data are derived from male rats of one age group only. Previously, a difference of *Bdnf* expression was found between the female and male FSL after escitalopram treatment.^66^ There is also report showing increased proinflammatory cytokine production in healthy elderly people.^67^ (4) We did not investigate DNA methylation within the promoter of pri-let-7i in FSL-runners, but only histone H3 and H4 acetylation. As reported by O'Hara *et al.*, DNA methylation was not detectable in the pri-let-7i promoter region in a cell line with high let-7i expression.^49^ However, DNA methylation variability in the pri-let-7i promoter of the rat PFC cannot be ruled out. (5) The correlation of gene expression to the time spent on wheel running or quantitative depressive behavior in individual rats was not assessed due to incomplete data. Behavior test was not performed on the FSL runners in this particular study, however, a similar wheel-running protocol had antidepressant-like effect in the FSL tested using forced swimming test.^42^ (6) We did not have the possibility to analyze *Il6* and let-7 in specific cell types in this experiment. However, *Il6* and let-7 are expressed throughout the central nervous system, including both neuron and glia cells.^28, 68, 69^

## Conclusion

We provide results demonstrating for, we believe, the first time that elevated proinflammatory *Il6* in the depressed brain is associated with let-7 deficiency. We show that the low levels of let-7 may be a result of disturbed LIN28B-mediated miRNA biogenesis and DROSHA dysregulation. Physical activity was found to normalize the *Il6* and let-7 levels through epigenetic regulations upstream primary miRNA transcription. Our results warrant further studies on let-7 regulation in depression. As modulators of IL-6 have been suggested to have a therapeutic potential for treatment-resistant depression,^8^ further investigation of let-7 is indicated to test this hypothesis.
